# Distributed Fiber-Optic Sensors for Vibration Detection

**DOI:** 10.3390/s16081164

**Published:** 2016-07-26

**Authors:** Xin Liu, Baoquan Jin, Qing Bai, Yu Wang, Dong Wang, Yuncai Wang

**Affiliations:** Key Laboratory of Advanced Transducers and Intelligent Control Systems, Ministry of Education, Taiyuan University of Technology, No. 79 Yingzexi Street, Taiyuan 030024, China; liuxin0924@link.tyut.edu.cn (X.L.); jinbaoquan@tyut.edu.cn (B.J.); baiqing0122@link.tyut.edu.cn (Q.B.); wangyu@tyut.edu.cn (Y.W.); wangdong@tyut.edu.cn (D.W.)

**Keywords:** vibration detection, distributed fiber-optic sensor, interferometric sensing technology, backscattering-based sensing technology, structural health monitoring, perimeter security

## Abstract

Distributed fiber-optic vibration sensors receive extensive investigation and play a significant role in the sensor panorama. Optical parameters such as light intensity, phase, polarization state, or light frequency will change when external vibration is applied on the sensing fiber. In this paper, various technologies of distributed fiber-optic vibration sensing are reviewed, from interferometric sensing technology, such as Sagnac, Mach–Zehnder, and Michelson, to backscattering-based sensing technology, such as phase-sensitive optical time domain reflectometer, polarization-optical time domain reflectometer, optical frequency domain reflectometer, as well as some combinations of interferometric and backscattering-based techniques. Their operation principles are presented and recent research efforts are also included. Finally, the applications of distributed fiber-optic vibration sensors are summarized, which mainly include structural health monitoring and perimeter security, etc. Overall, distributed fiber-optic vibration sensors possess the advantages of large-scale monitoring, good concealment, excellent flexibility, and immunity to electromagnetic interference, and thus show considerable potential for a variety of practical applications.

## 1. Introduction

Vibration is a common phenomenon in nature and vibration monitoring and analysis technology is of significant importance in scientific measurements and engineering applications. Accurate measurement and monitoring of vibration is crucial for detection of the abnormal events and pre-warning of infrastructure damage [1]. Various vibration sensors are already available and are mainly based on piezoelectric [2,3], magnetostrictive [4], capacitive [5], and inductive [6] technologies, etc. However, these traditional vibration sensors suffer from electromagnetic (EM) interference, which presents difficulty for applications in harsh environments. In addition, the short monitoring distance and high maintenance cost means they do not meet the actual needs of modern engineering measurement. As the field of vibration sensor has advanced, strong interests exist for new vibration sensors replacing the traditional sensors to improve cost-effectiveness and the immunity to EM interference.

On the other hand, optical fibers have attracted a significant amount of research attention in a wide range of applications during the last several decades due to the outstanding advantages of light weight, flexible length, high accuracy, signal transmission security, easy installation, corrosion resistance, and immunity to EM interference [7,8]. These characteristics render them attractive for use in harsh environments where the application of traditional sensors is severely limited [9]. Besides their wide usage in telecommunications areas, optical fibers have shown huge application potential in fiber sensors in recent decades. The high sensitivity to changes in external physical quantities, such as temperature, strain, and vibration, makes optical fibers suitable for sensing purposes [10]. Generally, the operating principle of a fiber-optic vibration sensor is based on the modulation of the light property, such as intensity, phase, polarization state, or light frequency, which is induced by the applied external vibration.

Up to now, fiber-optic vibration sensors mainly consist of point [11,12,13,14], quasi-distributed [15], and distributed sensors [16,17]. Several schemes of point sensors including fiber Bragg grating (FBG), Fabry–Pérot [18,19], self-mixing [20], and Doppler vibrometry [21,22] are deployed for vibration measurement. Among them, FBG vibration sensors have become a fast-developing scientific research field owing to intrinsic advantages such as low noise, good embeddability, and ability to be easily multiplexed to construct a distributed sensor array [23,24]. Based on the FBG sensing principle, many investigations are applied to the measurement of vibration. For instance, a low-cost optical accelerometer system, capable of detecting frequencies up to 45 Hz, was reported [25]. Then, a compact FBG diaphragm accelerometer based on L-shaped rigid cantilever beam was proposed and dynamic vibration measurement showed that a frequency response range of 0–110 Hz was obtained [26]. Afterwards, a novel FBG accelerometer based on a diaphragm was presented, which offered a linear response over a wider frequency range from 10 to 200 Hz [27]. In addition, with the development of vibration sensors, several schemes of FBG sensors are applied to the field of quasi-distributed vibration measurements [28,29]. However, all of the aforementioned methods require prior knowledge of the potential vibration positions, which is not suitable for some practical applications. Clearly there is a need for a technique that allows continuous vibration measurements to be captured in real time over lengths from a few meters to tens or hundreds of kilometers. Fortunately, the advent of distributed fiber-optic vibration sensors makes this possible.

Distributed fiber-optic vibration sensing technology is able to provide fully distributed vibration information along the entire fiber link, and thus external vibration signals from an arbitrary point can be detected and located. Compared with point and quasi-distributed vibration sensors, which can only be used individually on a small scale and often have poor concealment, distributed fiber-optic vibration sensors inherit the advantages of general fiber sensors and offer clear advantages such as light weight, large-scale monitoring, good concealment, excellent flexibility, geometric versatility of optical fibers, quick response, system simplicity, immunity to EM interference, high sensitivity, accurate location, etc. [30,31,32].

In the past decades, distributed fiber-optic vibration sensing technology has received great attention and experienced an explosive growth. Up to now, distributed fiber-optic vibration sensors mainly include interferometric sensors and backscattering-based sensors. Various interferometric sensors have attracted a significant amount of research attention and are widely investigated. Of these, the Sagnac interferometer [33], the Mach–Zehnder interferometer (MZI) [34], and the Michelson interferometer (MI) [35] have made a significant entrance in the fiber-optic vibration sensor panorama. Another distinguished technique is backscattering-based sensors, which are mainly based on optical frequency domain reflectometer (OFDR) and optical time domain reflectometer (OTDR) such as the phase-sensitive OTDR (Φ-OTDR), polarization-OTDR (POTDR), Brillouin optical time domain analysis (BOTDA), or Brillouin optical correlation domain analysis (BOCDA). Particularly, there has been a considerable interest in the development of OTDR-based sensors during the last several decades; some of them, such as the Φ-OTDR [36] configurations, have attracted more and more research attention and experienced tremendous advancement in recent years. Generally, OFDR is used for stationary measurement, and recently some schemes for dynamic sensing have been proposed.

This review paper is an attempt to summarize the evolution of the distributed fiber-optic vibration sensors, so that the advantages, limitations, and applications of this technology will be demonstrated and revealed to the readers. In Section 2, the distributed fiber-optic vibration sensing technologies, ranging from interferometric sensing to backscattering-based sensing, are described. Their operation principles are presented and recent research efforts are also included. Section 3 is given to some typical applications of distributed fiber-optic vibration sensors. Finally, conclusions are drawn in Section 4.

## 2. Distributed Fiber-Optic Vibration Sensing Technology

### 2.1. Interferometric Sensing Technology

**Figure 1:**
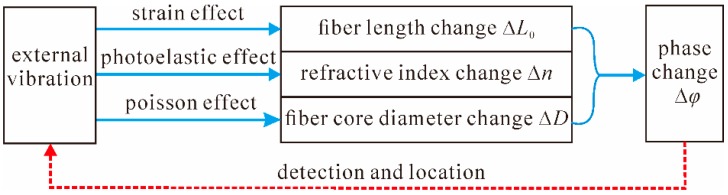
Schematic diagram of the detection principle of distributed fiber-optic interferometric vibration sensors.

In the past decades, distributed fiber-optic vibration sensors based on interferometric sensing technology have received an extensive amount of research attention because of the advantages of low cost and high sensitivity. For distributed fiber-optic interferometers, the vibration information is acquired on the basis of optical wave phase change detection. Generally speaking, when light passes through a single-mode fiber (SMF) whose length is *L*_0_, the phase delay of the exit light wave *ϕ* can be expressed as [37]:

*ϕ* = (2π*n*/λ_0_)*L*_0_ = *βL*_0_ (1)

where *λ*_0_ is the wavelength in a vacuum, *n* is the refractive index of the fiber core, and *β* is the propagation coefficient in the fiber. As schematically shown in Figure 1, when an external vibration signal is applied on the optical fiber, a pressure *P* will be generated at the corresponding position, which results in changes in the fiber length, the refractive index of the fiber core, and the core diameter due to the influence of strain effect, photoelastic effect, and Poisson effect, respectively, such that [37]:

∆*ϕ* = *β*∆ *L*_0_ + *L*_0_∆*β* = *βL*_0_ (∆*L*_0_/*L*_0_) + *L*_0_ (∂*β*/∂*n*)∆*n* + *L*_0_ (∂*β*/∂*D*)∆*D* (2)

where ∆*n* is the change of refractive index of the fiber core, *D* is the core diameter, and ∆*ϕ* is the phase change caused by ∆β and ∆*L*. Eventually, Equation (3) can be achieved on the basis of Equation (2), such that [37]:

∆*ϕ* = β*L*_0_*P*/*E* (1 − 2*μ*)[1/2 *n*^2^ (*P*_1_ + 2*P*_2_) − 1] (3)

where *E* is the young modulus, *μ* is the Poisson ratio, and *P*_1_ and *P*_2_ are only associated with *P*. As shown in Equation (3), the phase change is proportional to the external pressure *P* under the condition that the other parameters of the optical fiber remain constant. Therefore, the modulation effect of external pressure *P* can be manifested through measuring the optical wave phase change ∆*ϕ*.

**Figure 2:**
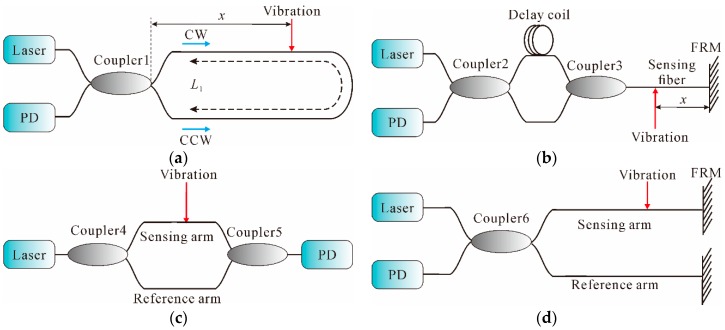
Distributed fiber-optic vibration sensors based on the interferometric technology: (**a**) Sagnac sensors of the loop configuration; (**b**) Sagnac sensors of the in-line configuration; (**c**) MZI sensors; (**d**) MI sensors.

In general, distributed fiber-optic interferometric vibration sensing technology mainly includes Sagnac (loop configuration and in-line configuration), MZI, and MI, whose basic configurations are all schematically shown in Figure 2.

#### 2.1.1. Sagnac

On the basis of the fiber gyroscopes, Udd, Kummer et al., further exploited the Sagnac technology in the field of distributed fiber-optic sensing [38,39]. In its most basic form, as shown in Figure 2a, the output of the laser is split into two beams through a 50:50 coupler and directed into two propagating directions, clockwise (CW) and counterclockwise (CCW) light waves, around the sensing fiber ring. Therefore, the field strengths of the CW and CCW waves are equal. Then, the two counter-propagating waves are recombined and converted into electrical outputs by a photodetector (PD). When an external vibration is applied at a distance *x* from the left end of the fiber on the sensing loop, the phase of the CW light is modulated earlier than the CCW light due to the non-central location of the vibration. Therefore, a phase difference between the two returning counter-propagating waves is induced, which results in a change of the interference intensity at the output of the loop. According to [40], the vibration location can be determined, as shown in Equation (4):

*x* = [*L*_1_ − N*c*/(n*f*_s,null_)]/2 (4)

where *L*_1_ is the length of the fiber, *c* is the velocity of light, n is the refractive index, *f*_s,null_ is the frequency of the first null, and N is an integer. Due to the merits of zero-path-length mismatched characteristic, Sagnac is insensitive to slowly varying fluctuations. In addition, Sagnac is of low expectation of light source, which makes it more practical and cheaper [41]. Therefore, many investigations concerning the Sagnac interferometer are carried out. In order to improve this technology, a variety of configurations have been proposed, including adopting a variable-loop Sagnac [42], incorporating dual-wavelength light sources [43], using twin Sagnac interferometers [44], adding an additional sub-loop [45], utilizing the configuration of a polarization-maintaining (PM) fiber [46], etc.

The abovementioned Sagnac interferometers are ordered in loop configuration, hence half of the fiber length needs isolation from the physical field in practical measurement, which increases the cost and prevents its further application, especially in the field of long-distance monitoring. Therefore, in order to improve the adaptation of the layout in field applications, a distributed fiber-optic system using an in-line configuration instead of the loop type was reported [47]. Its basic scheme is shown in Figure 2b. In the configuration, two couplers are employed. The two terminals of Coupler 2 are connected to a laser and a PD, respectively. One terminal of Coupler 3 is connected to the sensing fiber before the Faraday rotator mirror (FRM), which is connected at the end of the sensing fiber to compensate for the polarization induced signal fading. According to [48], the vibration location can be expressed as:

*x* = *c*/(4*nf*_s,null_) (5)

Thus, the vibration position can be located by in-line Sagnac interferometers. Although the limitation of central point detection failure in the loop configuration is eliminated, it is not sensitive enough for low-frequency vibration detection due to the output characteristics of the Sagnac interferometer.

In summary, these sensors are able to locate the vibration position when only one vibration is applied on the sensing fiber at one time. However, once multiple vibrations are applied on the sensing fiber simultaneously, the Sagnac interferometers locate neither vibration since the regularity of null frequencies disappears. Hence, some studies focusing on the problem of multiple vibrations were carried out. For example, a dual Sagnac loop system utilizing a wavelength division multiplexer (WDM) and fast Fourier transform (FFT) was proposed [49]. The experimental results showed that sinusoidally varying phase disturbances of 0.025 of a radian amplitude over 40 km sensing loop were located, with a 100 m positional sensitivity. However, this technique needed complicated configuration and signal processing methods, and crosstalk was inevitable. Later, a modified Sagnac distributed sensor, which was able to locate a disturbance synchronously acting on two points of a sensing fiber, was proposed. Experimentally, disturbance position was determined with an average error of 59 m over a total fiber length of 25 km [50]. Then, an improved algorithm of twice FFT, which focused on the overall spectrum, was presented for multi-point intrusion location [51]. Two vibrations at different positions responded as two separate peaks in the final location curve. The experimental results showed that a location error less than 100 m for single vibration was achieved over a total fiber length of 41 km. Moreover, the locating ability for multiple vibrations was also demonstrated. However, this method was not without flaws; some difficulties, such as poor signal-noise-ratio (SNR) and uncertain practical feasibility, still exist. Recently, Wang et al. [52] paid attention to these issues and improve the twice-FFT algorithm further. With the help of autocorrelation, peak concentration was improved effectively. In the experiment, a piezoelectric tube (PZT) was utilized to produce the ultrasonic, while knocking was also applied uniformly at a different position. Experimentally, the location performance decayed to 57 m and 181 m when pulse and ultrasonic were applied at 100 km and 90 km simultaneously.

#### 2.1.2. MZI

The use of MZI for the measurement of vibration has also been extensively investigated owing to its simple structure and straightforward locating method, and hence, MZI is considered as a cost-effective approach to produce distributed fiber-optic sensing [53]. In its most basic form, as shown in Figure 2c, light from a laser is split into two separate paths through Coupler 4. The external vibration is applied on the sensing arm while the reference arm is shielded from the changes of external vibration. Thus, the changes of length and refraction index cause phase modulations between the sensing arm and the reference arm. Then, the phase modulations are converted into intensity modulations through Coupler 5 and then detected by a PD [54].

**Figure 3:**
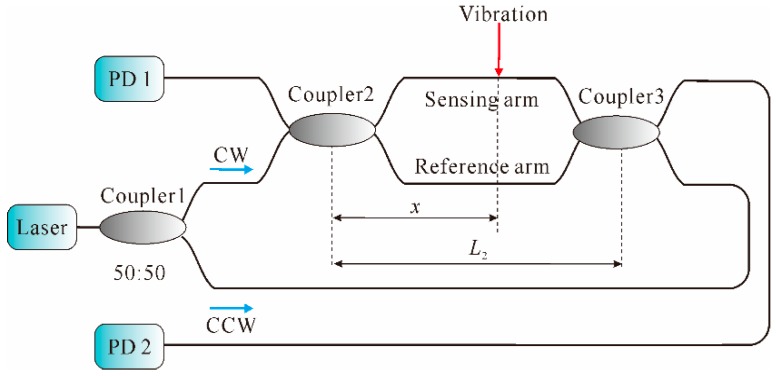
Schematic diagram of DMZI vibration sensors.

However, single MZI could merely sense external vibration signals but is not able to locate them. Usually, dual MZI (DMZI) architecture [55,56,57] is more widely investigated for vibration locating; its basic schematic diagram is shown in Figure 3.

As illustrated in Figure 3, through Coupler 1, light from the laser is equally split into two paths: CW and CCW light waves, which simultaneously transmit in both the sensing arm and the reference arm. Once an external vibration is applied on the sensing arm, a phase change between the two arms is thereby produced at the corresponding point. Then, the light waves transmit to the two ends of the optical fiber and interfere at both ends. Finally, the signals are received by PD 1 and PD 2 from the CCW and CW light wave. Consequently, the vibration point where the external vibration occurs can be obtained by [31]:

*x* = *L*_2_ − *c*∆*t/*(2*n*) (6)

where *x* is the distance from the point to Coupler 2, *L*_2_ is the length of the interference region, and ∆t is the time delay, which can be obtained by means of the correlation operation [58]. Since the CW and CCW lights are emitted from the same laser and affected by the same vibration, the two interfering signals detected by PD1 and PD2 are of strong correlativity. Therefore, time delay ∆t can be calculated by locating the peak position of the cross-correlation function between the two detected signals. Thus, external vibration can be eventually located by using the DMZI system. In addition, Sun et al. proposed a ring MZI architecture for distributed fiber-optic vibration sensing [59]. The vibration signal was successfully located and recovered by adding two symmetric 3 × 3 couplers and two circulators into the ring MZI. In addition, the sensor was able to detect multiple vibrations at different positions by using an automatic detecting scheme. The experimental results showed that a spatial resolution (SR) better than 38 m over a 1.01 km fiber length was achieved. However, for both the DMZI and ring MZI sensing systems, accurately calculating the time delay ∆t is a critical problem, since it is directly associated with the location accuracy. At present, the prevalent technique for obtaining the vibration position is time delay estimation (TDE) [60,61], which is mainly based on the cross-correlation function. For example, an ultra-long distributed fiber-optic intrusion detection system employing a cross-correlation algorithm was proposed, of which the location precision was about 160 m over a total sensing fiber length of 112 km [31]. Later, a positioning error reduction technique, in which a high-pass filter was utilized to reshape the original power spectrum, was proposed and a lower mean square error of the cross-correlation-based positioning algorithm was obtained [62]. However, this method was vulnerable to slow time-varying phase fluctuation, which was normally solved by utilizing the phase generation carrier (PGC) technique [63]; however, this would increase the complexity and cost in practical applications. Afterwards, an improved positioning algorithm for a DMZI sensing system employing zero-crossing method was proposed, which can improve the positioning accuracy with a positioning error of ±20 m [64]. Recently, a Lissajous figure based location method was proposed [65]. The experimental results showed that the maximal location error was 75 m over a 2 km fiber length.

#### 2.1.3. MI

**Figure 4:**
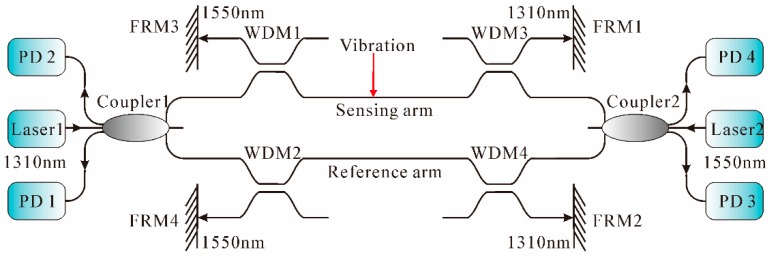
Schematic diagram of dual MI vibration sensors, adapted from Hong et al. [70].

MI is also a well-established and widely used technique. The typical MI architecture used as a vibration sensor is shown in Figure 2d. Through Coupler 6, light from the laser is split into two beams, which are reflected by FRMs and recombined in Coupler 6. When the optical path difference between these two beams falls within the coherence length of the laser, a fringe pattern is thereby produced. The zero-order fringe corresponds to the exact optical path matching these two beams [66]. On the basis of the basic MI architecture, many efforts have been made to improve the performance of vibration measurement, using different schemes such as dual output grating [67], step interferometry [68], unbalanced arms [69], etc. In 2010, an optical vibration sensor based on dual MIs was proposed [70]; the schematic is illustrated in Figure 4.

As shown in Figure 4, the system consisted of two MIs operating at different wavelengths (1310 nm and 1550 nm), respectively. The light from laser 1 was split into three parts by Coupler 1 and guided by WDM1 and WDM2 to the sensing arm and reference arm. Then, the two signals were guided by WDM3 and WDM4 to FRM1 and FRM2, which would reflect the 1310 nm signals back to Coupler 1, and interfered with each other. The two outputs of Coupler 1 were detected by PD 1 and PD 2. Another MI operating at 1550 nm operated similarly in the opposite direction. The experimental results showed that a SR of 51 m was achieved over a 4012 m sensing fiber length. The positioning principle of external vibration signals was based on the null frequency. However, the null frequency was often unclear because of the Rayleigh scattering and superposition of spectrums of interference signals of light echo and reflected light by FRM. Recently, a simple approach of adding a phase modulator at the end of the sensor arm to improve the positioning accuracy was reported in [71]. The experimental results showed that the null frequency became clearer and the maximum positioning error was in the range of 50 m.

#### 2.1.4. Combinations of Sagnac, MZI, and MI

**Figure 5:**
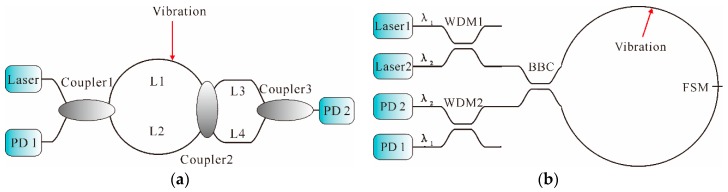
The combination of interferometric techniques: (**a**) Sagnac–MZI, adapted from Chtcherbakov et al. [73]; (**b**) Sagnac–MI, adapted from Spammer et al. [77].

Various architectures based on the combination of Sagnac, MZI, and MI have been proposed and widely discussed. The first reported was a two-interferometer structure incorporating an MZI with a Sagnac interferometer [72]. An 1150-nm HeNe laser was used in the sensor to provide the required coherence length. The output of Sagnac interferometer was proportional to the product of the rate of phase change and the distance of the perturbation (temperature change) from the center of the fiber loop, while the output of the MZI was merely proportional to the phase shift induced by the perturbation. This sensor detected disturbances with a SR of approximately ±20 m over a 200 m fiber length. One problem with this architecture was the trade-off of the required light source coherence between Sagnac and MZI. Then, Chtcherbakov et al. proposed a modified Sagnac–MZI distributed fiber-optic sensor using the arrangement shown in Figure 5a. On one side, the Sagnac portion was composed of a laser, L1, L2, Coupler 1, and PD 1. On the other side, a laser, L1 and L4 (the first arm), L2 and L3 (the second arm), and PD 2 comprised the MZI portion. The experimental results showed that the measurement accuracy was approximately 5 m over a sensing fiber length of 200 m [73]. Then, a distributed fiber-optic intrusion detection sensor system based on Sagnac–MZI that employed the hybrid time/wavelength division multiplexing (TDM/WDM) architecture was proposed [74]. Afterwards, a low-coherence triple detection fiber differential interferometer, based on an MZI and Sagnac hybrid interferometer, with adjustable measurement range was presented in [75,76].

Also, several interferometer combinations of Sagnac and MI are reported. A dual wavelength, merged Sagnac and MI system, as shown in Figure 5b, was proposed with a broad-band source for optimal operation [77]. It was composed of two light sources of different wavelengths (λ_1_, λ_2_), a broadband coupler (BBC), a frequency-selective mirror (FSM), and two WDMs. The FSM was transparent at wavelength λ_1_ and was highly reflective at wavelength λ_2_. Thus, Sagnac and MI were constructively formed. The signals of the two interferometers were directed to the corresponding PDs by the WDM2. If an external vibration was applied on the sensing fiber, the phase difference between the two beams in the MI was proportional to the phase change induced by the vibration. At the same time, the phase difference between the two counter-propagating beams in the Sagnac was proportional to the product of the rate-of-change of the phase and the distance of the vibration from the center of the Sagnac loop. Afterwards, a novel signal processing and localization scheme was presented to further improve the performance of Sagnac–MI sensors [78].

### 2.2. Backscattering-Based Sensing Technology

When an optical wave propagates along a fiber, both elastic scattering such as Rayleigh scattering and inelastic scattering such as Brillouin scattering are generated. The Rayleigh scattering is produced due to the microscopic variations in the density of optical fiber, while the Brillouin scattering is caused by the scattering of sound waves [79]. Generally, the travelling light scatters in all directions; a small portion of the light that propagates backwards is of significant interest and effectively exploited in the OTDR system for nondestructive fiber attenuation characterization and measurement, such as splice loss, connector insertion loss, micro-bending loss, as well as fiber and cable length, etc. [80].

**Figure 6:**
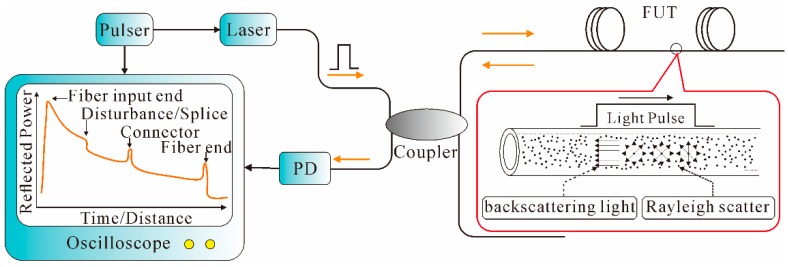
Typical OTDR setup and output waveform, adapted from Juarez [82].

OTDR, which was initially demonstrated by Barnoski and Jensen in 1976 [81], is typically composed of a pulser, a laser, a directional coupler, a PD, and a signal processor. As schematically illustrated in Figure 6, the laser pulse is launched into the fiber under test (FUT) through the directional coupler. The laser is fired when a trigger pulse that simultaneously triggers an oscilloscope is received. As the probe pulse propagates along the FUT, the Rayleigh backscattering light is collected by the PD via the directional coupler. Finally, a continuous waveform from all points along the fiber is thus generated in the signal processor. In addition, due to the presence of Fresnel reflection, abrupt peaks and dips are thereby formed at the corresponding position of splices, connectors, etc. [82].

In the OTDR system, the distance to the point of reflection is determined by the time delay between the launched light pulse and the corresponding Rayleigh backscattering signal, which can be expressed as:

*l* = 0.5*cτ/n*_g_ (7)

where *l* is the distance to the point of reflection, and *n*_g_ is the group refractive index of the fiber. The SR, ∆*z*, can be expressed as:

∆*z* = 0.5*cT*_p_*/n*_g_ (8)

where *T*_p_ is the pulse width. The reflected backscattering power *P*_s_ from a distance *l* can be calculated from Equation (9) [82]:

*P*_s_ = 0.5*Fα*_s_·*v*_g_·*τP*_i_·*exp*(−2*αl*) (9)

where *F* is the capture coefficient, *α*_s_ is the Rayleigh scattering coefficient, *v*_g_ is the group velocity in the fiber, *P*_i_ is the input power, and *α* is the fiber loss coefficient. Thus, the reflected backscattering power *P*_s_ decreases exponentially with respect to time/distance along the fiber due to the presence of transmission loss. Therefore, by monitoring the reflected backscattering power, the distribution of the attenuation characterization along the whole fiber link can be obtained. OTDR possesses the merits of distributed measurement capability; however, when it is used for distributed sensing, the sensitivity is very low. Hence, many efforts have been made to fulfill distributed vibration sensing based on the conventional OTDR technology, such as Φ-OTDR, POTDR, BOTDA, etc.

**Figure 7:**
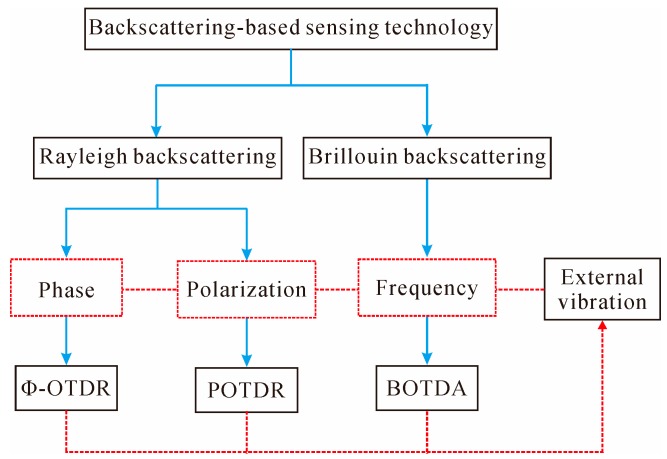
Schematic diagram of the detection principle of backscattering-based vibration sensors.

As shown in Figure 7, the backscattering-based sensors detect external vibration signals by measuring the optical characteristics of the Rayleigh or Brillouin backscattering light, such as the light phase (Φ-OTDR), polarization state (POTDR), or frequency (BOTDA). Their operating principles are detailed in the following sections.

#### 2.2.1. Φ-OTDR

The Φ-OTDR technique was first proposed in 1993 by Taylor et al. for monitoring intrusion events by detecting the intensity changes of interferometric light [83]. Essentially, Φ-OTDR has the same setup as a conventional OTDR except that the light source is a highly coherent laser with narrow line width. Conventional OTDR uses broadband light source and therefore can only measure intensity variations along the fiber. In contrast, Φ-OTDR uses a highly coherent laser, hence the output is modulated in the form of a speckle-like profile [84] owing to the coherent interaction of numerous scattering centers within the pulse duration. When an external vibration, such as the intrusion signal, is applied on a certain part of the sensing fiber, the refractive index and/or length of the fiber will change at that position, resulting in a localized phase change in the light wave. Therefore, the light intensity will change at a time corresponding to the location. Therefore, the vibration can be detected by subtracting the coherent traces before and after a vibration event.

**Figure 8:**
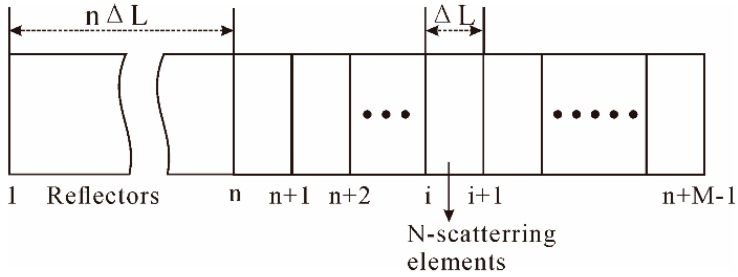
Discrete model of Rayleigh backscattering light, adapted from Park et al. [85].

Figure 8 illustrates a discrete model of Rayleigh backscattering light [85], where the backscattering process can be briefly described as a series of reflectors. Assume that each reflector is composed of N independent Rayleigh scattering elements, randomly distributed in a particular length of fiber L. The interference field of backscattering light from M reflectors within a pulse width of MΔL at distance nΔL can be determined from Equation (10):

(10) $$E_{b}(n\Delta L)=E_{0}\overset{n+M-1}{\underset{i=n}{\sum }}P_{i}r_{i}\exp(j(\emptyset_{i}+\theta_{i})e^{-\alpha i\Delta L})$$

where $E_{0}$ is the field amplitude of incident light, $r_{i}$, $\emptyset_{i}$ and $P_{i}$ are the random reflectivity, phase, and random polarization of the i-th reflector, respectively, α is the power attenuation coefficient, ΔL is the unit fiber partition length, and $\theta_{i}$ is the phase change induced by the vibration signal in the i-th reflector. Thus, the interference field of backscattering light at a time corresponding to the location of the vibration is changed. Moreover, like the conventional OTDR, the SR in Φ-OTDR system also depends on the pulse width, as presented in Equation (8).

**Figure 9:**
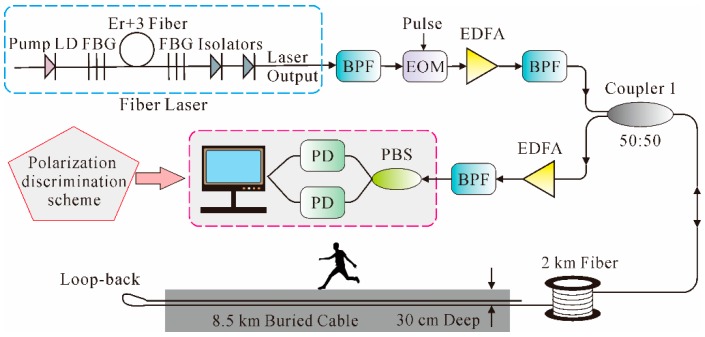
Field tests setup for the Φ-OTDR intrusion detection system, adapted from Juarez et al. [89].

Φ-OTDR technology is a typical distributed fiber-optic sensor to detect and locate vibrations along the fiber, and has received considerable research attention since it was proposed in 1993. For instance, Park demonstrated the effectiveness of Φ-OTDR with a semiconductor ring laser with 50 kHz line-width and acousto-optic modulator (AOM); the sensing range was 6 km and SR was 400 m [86]. In 2005, Juarez et al. designed a distributed sensor system for detecting and locating intruders based on the Φ-OTDR technology by using a continuous-wave Er:fiber Fabry–Pérot laser with a narrow (3 kHz) instantaneous line width and low frequency drift (few kilohertz per second); the SR is 1 km over a sensing range of 12 km [87]. Later in the same year, they proposed a polarization discrimination scheme and successfully improved the ability to detect an intruder by providing two statistically independent channels, which reduced the possibility of a missed intruder. In addition, an improved version of a fiber laser with increased frequency stability, optical output power, and coherence was utilized. Test results showed that the detection of intruders on foot as far as 4.5 m from the cable line was consistently obtained with a monitored length of 12 km [88]. Later, in 2007, field tests of the sensor system were performed in desert terrain. The sensor consisted of 8.5 km of two-fiber, 4.5 mm outdoor cable buried in desert soil in a 30 cm deep, 76 cm wide triangular trench filled with loose sand. The sensing range was improved to 19 km by splicing the two fibers at the end of the cable in a loop-back arrangement and adding a 2-km fiber which was thermally and acoustically insulated to the input side. The arrangement is shown in Figure 9 [89].

As illustrated in Figure 9, light from the fiber laser passed through a bandpass filter (BPF) to remove spontaneous emissions. Then, through an electro-optic modulator (EOM), narrow light pulses were produced and subsequently amplified by an erbium-doped fiber amplifier (EDFA), the output of which was filtered by a second BPF and finally launched into the FUT via Coupler 1. Then, through the polarization beam splitter (PBS), the orthogonal polarizations of the backscattering light were monitored with separate receivers. The experimental results showed that high sensitivity and consistent detection of intruders on foot and of vehicles traveling down a road near the cable line was realized over a cable length of 8.5 km and a total fiber path of 19 km.

**Figure 10:**
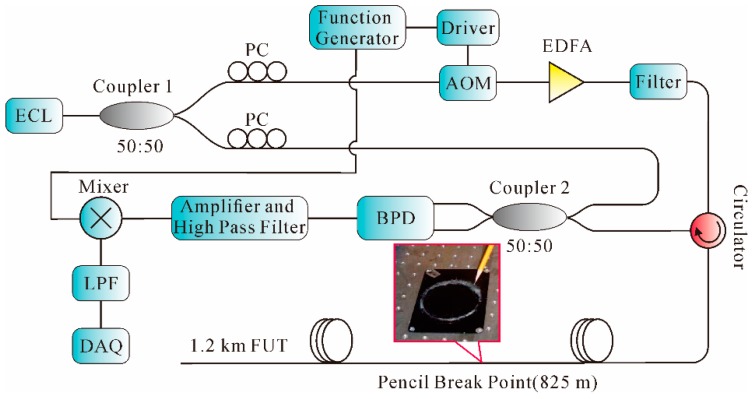
Experimental setup for coherent Φ-OTDR, adapted from Bao et al. [90].

With the development of Φ-OTDR, the mere detection of vibration position cannot satisfy the practical needs. The abovementioned Φ-OTDR was utilized to detect the intruder without giving the frequency information. Therefore, studies in regard to frequency information of vibration signals were then undertaken. In 2010, a Φ-OTDR system based on coherent detection was proposed by Bao et al., using the arrangement shown in Figure 10 [90].

As shown in Figure 10, a 1548.2-nm optical waveform from the external cavity laser (ECL) with narrow line width of 20 kHz and low frequency drift of 5 MHz was split into two parts through Coupler 1. One part was modulated into narrow pulses with a repetition rate of 10 kHz through AOM. Then, after being amplified by EDFA and filtered by the FBG filter, the light pulses were launched into a 1.2 km FUT via a circulator. Another part was used as a local oscillator (LO) with a polarization controller (PC), which was then combined with a backscattering Rayleigh signal via Coupler 2. By using a balanced PD (BPD), the amplitude of backscattering signal was improved 3 dB. After it went through a mixer with a normal sine waveform of 200 MHz, the signal passed through a low-pass filter (LPF) and was sampled by a data acquisition (DAQ) card. Instead of using the separating averaging method, which required a great number of waveforms to obtain a high SNR, moving averaging and moving differential methods were introduced in the system to increase the vibration frequency response. In the experiment, broadband acoustic frequency components generated by pencil-break vibration were measured and identified in location. The SR was 5 m and the highest frequency response was 1 kHz by using heterodyne detection and signal processing of the moving averaging and moving differential methods. In the next year, Bao et al. further developed an Φ-OTDR system based on all polarization-maintaining (PM) configurations. The state of the polarization (SOP) remained constant throughout the system and thus the intensity fluctuation noise caused by the polarization state change was reduced. The experimental results showed that the detectable frequency response was increased to 2.25 kHz, with an SR of 1 m over a total fiber length of 200 m [91]. However, this PM configuration system was expensive for practical application.

Also, an Φ-OTDR scheme that was able to detect ultrasonic waves was presented [92]. A high extinction ratio (ER) between pump pulses was achieved by utilizing a semiconductor optical amplifier (SOA), which contributed to decrease the intra-band noise greatly and allow frequency measurements in the limits set by the flight time of the light pulses. The sensor was able to measure vibrations of up to 39.5 kHz with a 5-m SR over a range that could reach 1.25 km.

Then, a novel distributed vibration sensor with high frequency response and high SR based on the TDM scheme was proposed [93]. Vibration with frequency of 0.6 MHz with 1-m SR can be measured over a 680 m SMF. Afterwards, great attention was focused on by the sensing distance of Ф-OTDR system. Since the backscattering signal was decreased about tens of decibels in comparison to the forward propagating pulse power level, the received signal power level was usually very low, which limited the maximum achievable distance [94]. Valuable work had been carried out to improve the performance of Ф-OTDR. However, the trade-off among SNR, SR, and sensing range always existed in the Ф-OTDR system [95,96]. Thus, simultaneously achieving long distance, high SR, and high SNR in the Ф-OTDR system was a huge challenge. The normally used EDFA-based amplification method is limited by the peak power of probe pulses in order to suppress nonlinear effects in the fiber, like modulation instability [97].

**Figure 11:**
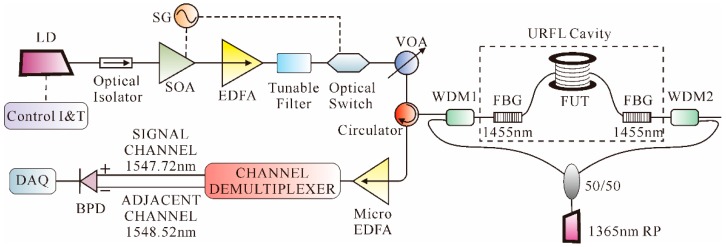
Experimental setup of Φ-OTDR using an URFL cavity, adapted from Martins et al. [99].

In [98], Martins et al. proposed a novel scheme, through which ultra-long distance and frequency information were simultaneously obtained. 1st-order bidirectional Raman amplification (BRA) was proposed and deployed in Ф-OTDR to extend the sensing range; for example, in [32], the sensing distance was improved to 62 km with 100 m SR. Afterwards, an optical switch was used to greatly decrease the intra-band coherent noise, which extended the sensing range significantly. The sensor was able to detect vibrations of 250/300 Hz at a distance of 125/110 km with an SR of 10 m and no post-processing. Later, an ultra-long Raman fiber laser (URFL) cavity was added to the system [99]; the experimental setup is shown in Figure 11.

As shown in Figure 10, a 1546-nm laser diode (LD), driven by a standard current and temperature controller, was utilized as the laser source. Then, after an optical isolator, the light wave entered a SOA, which was driven by a waveform signal generator (SG), to achieve high ER of the pulses. An EDFA was used to boost the power of the input pulses. Afterwards, a tunable filter was utilized to remove the ASE. Then, after a tunable filter, an optical switch that was also driven by the SG synchronized with the pulse was used in order to further increase the ER. Then, light passed through a variable optical attenuator (VOA) to adjust the input power. The FUT was connected to the common ports of two WDMs. The input pulses were launched into the FUT through the 1550-nm port of the WDM1. Through a calibrated 50/50 coupler, the light from a 1365-nm Raman pump was split into two parts, which were respectively coupled into the 1310-nm port of the WDM1 and WDM2. Two 1455-nm FBGs with 0.5 nm width at half maximum and 80% reflectivity were placed in both ends of the fiber, thus creating the URFL cavity. Then, after being amplified by a micro-EDFA, the Rayleigh backscattering signal goes through a 100 GHz channel demultiplexer to filter out the channel of the signal and an adjacent channel, which were detected by a BPD and then sampled by a DAQ. The sensor was able to measure vibrations of up to 380 Hz over a distance of 125 km with an SR of 10 m. Afterwards, an Φ-OTDR architecture that combined heterodyne detection and 1st-order BRA was proposed to realize ultra-long and high sensitivity vibration sensing [100]; the experimental results showed that high SNR over a whole 131.5-km fiber with an SR of 8 m and a maximum detection frequency of 375 Hz was obtained.

**Figure 12:**
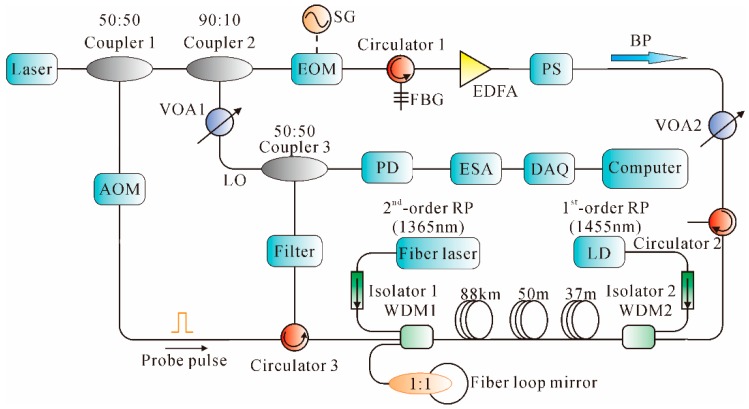
Experimental setup for ultra-long Φ-OTDR utilizing a novel hybrid amplification scheme, adapted from Wang et al. [101].

In [101], a novel hybrid amplification scheme was demonstrated to further improve the sensing distance by using the arrangement shown in Figure 12. The light source with an ultra-narrow-line width (100 Hz) laser operating at 1549.860 nm was utilized, which was equally split into two equal portions through Coupler 1. One portion was modulated into the probe pulses with 500 Hz repetition rate and 250 ns width by an AOM, while the other portion was further split into two portions by Coupler 2. The 10% part was used as the LO, while the 90% part was modulated by an EOM to generate two sidebands with suppressed carrier. The high frequency sideband was selected as the Brillouin Pump (BP) by an FBG. The BP was amplified by an EDFA, and then depolarized using a polarization scrambler (PS). After being mixed with the LO, the Rayleigh backscattering light was detected by a PD, analyzed by an ESA, sampled by a DAQ card, and finally processed in a computer. In the hybrid amplification scheme, 2nd-order RP was used to amplify the pulse along the first segment (88 km). Then, the second segment (88–138 km) was significantly boosted by the BP through the delivery of 1st-order RP. Finally, the probe pulse was amplified by the 1st-order RP within the last segment (138–175 km). The experimental results showed that the sensing range was extended to 175 km, with an SR of 25 m.

As we know, the signal processing scheme is critical in getting high-performance distributed vibration measurement. A wavelet technique, which was based on threshold wavelet coefficients of the noisy signal to remove the noise, was proposed. Experimental results showed that a vibration frequency as high as 8 kHz can be detected with an SR of 0.5 m [102]. Afterwards, a continuous wavelet transform (CWT) method was proposed, which can simultaneously provide the frequency and time information of the vibration event. Distributed vibration measurements of 500 Hz and 500 Hz to 1 kHz sweep events over 20 cm FUT were demonstrated [103].

Extracting the information of the vibration signal accurately is of significant importance in the Φ-OTDR system. As mentioned before, the vibration location can be extracted by the moving differential method. Moreover, a two-dimensional (2D) edge detection method with the help of a Sobel operator was also introduced into the Φ-OTDR system to extract location information [104]. Generally, the information of vibration location and frequency were separately extracted. Firstly, the location was detected by the moving differential method or the 2D edge detection method. Secondly, the frequency information was obtained through FFT of the optical power variation in the located vibration position. However, the time domain information was lost when the FFT was performed. The Short Time Fourier Transform (STFT) [105] and the CWT were used in the time-frequency analysis, though they cannot give high-resolution results for both frequency and time domain simultaneously. Moreover, CWT algorithm was time-consuming for the signal processor. Afterwards, a Hilbert–Huang transform (HHT) [106], which has high frequency resolution but is much less time consuming, was introduced to the Φ-OTDR system. In [107], by applying a 1-D Fourier transform of a Rayleigh backscattering traces matrix in the Φ-OTDR distributed vibration sensing system, the simultaneous extraction of vibration location and frequency information with SNR and SR enhancement was realized. The SR was also improved from 5 to 3.7 m when the pulse duration was 50 ns. The maximum readable frequency of ∼18 kHz was achieved over a fiber length of 2.7 km.

As mentioned above, the vibration position and frequency had already been determined. However, for some applications, it is difficult to distinguish vibration behaviors if they are of the same frequency. Thus, in these situations, amplitude measurement is required. In recent years, research on vibration amplitude measurement in the Φ-OTDR system has been carried out. For instance, a phase-measuring Φ-OTDR system for quantitative dynamic strain measurement was presented with the help of an unwrapping algorithm. A dynamic strain with an amplitude of 200 nε at the end of a 24.61 km sensing fiber was successfully recovered [108]. Then, a system capable of simultaneously sensing strain and vibration was presented, with a 2 m SR, up to 1 kHz frequency range, and 10 nε strain resolution over a fiber length of 9 km [109].

#### 2.2.2. POTDR

**Figure 13:**
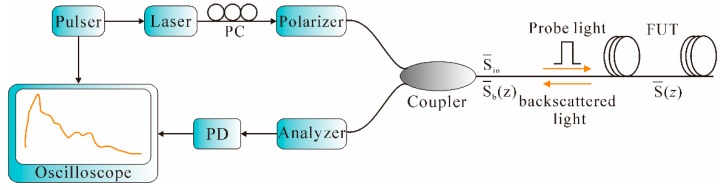
Basic arrangement of POTDR system.

POTDR, first proposed by A. J. Rogers in 1981, is based on the measurement of field distribution of the state of polarization (SOP) of the Rayleigh backscattering light [110]. The basic POTDR arrangement is shown in Figure 13.

As shown in Figure 13, the POTDR configuration can be constructed by adding several polarization control devices to the conventional OTDR. In the POTDR system, a polarizer is adopted to generate linearly polarized light, which is then launched into the FUT via the coupler. Usually, a PC is placed before the polarizer to adjust the SOP of the input optical fiber in order to achieve the optimal laser energy. Assume that S_in_, S (*z*), and S_b_ (*z*) are the SOP of the incident light, the light at the position *z*, and the Rayleigh backscattering light from *z*, respectively. This gives Equations (11) and (12) [111]:

S (*z*) = M·S_in_ (11)

S_b_ (*z*) = RM^T^RM·S (*z*) (12)

where M is the Mueller matrix from fiber initial end to *z* and R is the mirror matrix. Eventually, the SOP change of any point *z* along the fiber with respect to the initial end is thereby obtained. When external vibration is applied on a certain position on the FUT, the polarization is thereby modified and then detected by the analyzer via the coupler. Afterwards, the polarization information is converted to an electrical signal by the PD. Finally, a continuous waveform containing the polarization information of the Rayleigh backscattering light along the fiber is thus retrieved in the signal processor. Thus, the change of the SOP, and therefore the vibration, can be detected by the POTDR system. Like conventional OTDR, the vibration position and SR of the POTDR system can also be obtained from Equations (7) and (8), respectively.

In 2008, a spectrum density of the POTDR system for distributed vibration measurement was proposed [112]. The experimental results showed that over a total fiber link of 1 km, a 10 m SR and up to 5 kHz vibration frequency were detected without averaging. Also, two simultaneous vibrations with the same frequency could be successfully identified with a novel FFT spectrum analysis. However, the signal processing technique required to calculate the SOP evolution at each point of the fiber was of high complexity. Therefore, a novel kind of POTDR intrusion sensor with simple detection algorithm, which was based on the sliding standard deviation of the difference between two consecutive POTDR traces, was demonstrated [113]. Experimental results showed that an intrusion leading to a 1.5-cm fiber displacement could be detected, with an error on the intrusion localization of 5 m over a total fiber length of 472.5 m. Later, a distributed fiber strain and vibration sensor that effectively combined Brillouin OTDR and POTDR was proposed, a 10-m SR was realized over a 4-km sensing distance [114]. Afterwards, by using 2nd-order Raman amplification, a long-distance (106 km) POTDR fiber fence system was demonstrated [115]. Then, a POTDR combined with ultra-weak FBG (UWFBG) was demonstrated [116]. In the system, both the Rayleigh backscattering light and the UWFBG reflected signal consisted of the detected signal within one pulse at each moment, when the separation of UWFBGs was equal to the SR. Therefore, the amplitude of the detected signal was enhanced along the whole fiber, with an improvement of 11 dB in SNR and 9 dB vibration sensitivity.

In POTDR systems, multi-point vibration location has always been a knotty issue; usually only the beginning point of the vibration events can be determined because the backscattering light after the first vibration point is contaminated by the previous vibrations. Frequency spectrum analysis based on the FFT method [112] could generally identify different vibrations with a single frequency component or different frequencies. However, different vibrations with wide frequency range are common in practical applications, which makes the FFT analysis difficult to use in these situations. Some efforts have been made to improve the multi-point detection performance of the POTDR system [117,118], though multi-point vibration detection is a still challenging problem, which greatly restricts the practical applications of the POTDR system.

#### 2.2.3. BOTDA and BOCDA

When an optical wave propagates along a fiber, Brillouin scattering is also produced due to the scattering of sound waves moving in the opposite direction. Compared with Rayleigh scattering, Brillouin scattering is inelastic due to the characteristic of frequency shifts, namely Brillouin frequency shift (BFS). Usually, the BFS changes linearly with the external perturbation, which can be utilized for sensing purposes.

The BOTDA technique, which is based on stimulated Brillouin scattering (SBS), was first proposed by Horiguchi in 1989 for non-destructive measurement [119]. In the BOTDA system, two counter-propagating light waves, a pulsed pump and a continuous wave probe, interact along a sensing fiber. The weaker signal probe wave at a particular location may be amplified if the frequency difference between the counter-propagating lights is equivalent to the Brillouin frequency. Hence, the Brillouin signal of that part can be received again by adjusting the frequency difference between the pump light and probe light. Then, the Brillouin Gain Spectrum (BGS) can be recovered. Finally, frequency difference is thereby translated to the external measured parameter at each point along the sensing fiber.

This technique is traditionally limited to static measurements, such as strain or temperature, due to the long measurement time for scanning the probe frequency in order to obtain the BGS [120]. Recently, some studies on dynamic measurement with the BOTDA technique were successively carried out. For instance, a pulse-based BOTDA technique was proposed for distributed fiber-optic vibration sensing, by using a short pulse and the slope of the stimulated Brillouin spectrum. Using a 6.25 ns pulse, 160 MHz dynamic range was verified and different vibration frequencies were determined [121]. Afterwards, a Slope-Assisted (SA) BOTDA, which probed the fiber with a single frequency, allowed a single pump pulse to sample fast strain variations along the whole fiber length. By using a specially synthesized and adaptable probe wave, a fiber with an arbitrary distribution of the BFS can be interrogated. Strain vibrations of up to 400 Hz were demonstrated, simultaneously measured at two different positions acting on an 85-m fiber, having different static Brillouin shifts, with an SR of 1.5 m [122]. The SABOTDA technique retained the advantages of the conventional BOTDA while providing truly distributed sensing over hundreds of meters of fiber, having an arbitrary longitudinal Brillouin profile [123]. However, the measured strain vibration amplitude was limited to the extent of the linear section of the BGS slope. A new technique based on generation of frequency-agility microwave signal by an electric arbitrary waveform generator for fast implementation of BOTDA was proposed and demonstrated by dynamic sensing. By using a digital signal generator that enabled fast switching among 100 scanning frequencies, dynamic measurement of an SR of 1.3 m was realized over a 100-m fiber [124]. This system had the same problem as that in conventional BOTDA in terms of SR. Then, a modified differential pulse-width pair (DPP) scheme, which was utilized to improve the SR of fast BOTDA, was proposed. Two long pulses with different pulse-widths were used to form a differential double-pulse using pump pulses. The experimental results showed that a vibration frequency of up to 50 Hz was observed over a 50-m fiber with an SR of 20 cm [125]. Afterwards, a cyclic pulse coding technique based on quasi-periodic bit sequences was introduced to the BOTDA system for fast strain measurement. The coding gain was utilized to reduce the averaging number and then the measurement time. The experimental results showed that a minimum measurement time of 0.4 s was achieved along a 10-km sensing distance, with an SR of 1 m [126].

Additional, Hotate et al. developed another technique based on SBS, BOCDA, for distributed dynamic measurement [127]. In the BOCDA system, both the pump and probe waves are continuous light, propagating in opposite directions with the frequency offset ∆f sweeping around the BFS of a FUT. In order to localize the Brillouin interaction, both waves are frequency-modulated in a sinusoidal way at the frequency f_m_ with the modulation amplitude ∆f to generate a correlation peak (measurement position). The SR in BOCDA, ∆z, can be expressed as:

∆*z* = (*v*_g_·∆*v*_b_)/(2π·*f*_m_·∆*f*) (13)

where ∆v_b_ is the intrinsic line width of the Brillouin scattering. Thus, millimeter-order SR can be obtained with the BOCDA technique. For example, a BOCDA system with vibrations up to 200 Hz with 10 cm SR over a 20-m measurement range was reported [128]. Then, based on BOCDA, a distributed measurement system of 1.3 Hz vibration, with an SR of 80 cm over a 100-m fiber length was experimentally achieved [129]. However, due to the influence of the correlation peak characteristic, the measurement range l_m_ is usually limited, which can be expressed as Equation (14):

*l*_m_ = *v*_g_/2*f*_m_ (14)

It can be seen from Equations (13) and (14), under the conditions of a certain SR, in order to improve the measurement range, ∆*f* should be increased. However, if ∆*f* is larger than half of BFS, spectrum aliasing of the pump and probe will be generated, which eventually causes an increase in system noise. Therefore, the sensing distance is limited in the BOCDA system, though high SR can be obtained.

#### 2.2.4. OFDR

**Figure 14:**
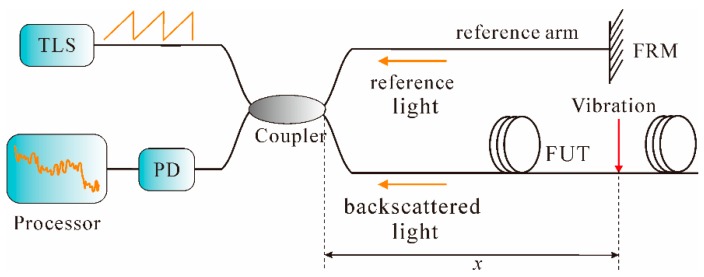
Basic arrangement of OFDR system.

The OFDR technique is based on swept-wavelength homodyne interferometry [130]. The basic OFDR arrangement includes a tunable laser source (TLS), an interferometer structure, a PD, and a signal processor, as shown in Figure 14.

As can be seen from Figure 14, in the OFDR system, a light wave from the TLS is linearly chirped and then split into two beams. One beam is launched into the FUT as the probe light to produce the Rayleigh backscattering light, while the other beam is chosen as the reference light for coherent detection [131]. As the laser frequency tuned, the interference fringes are generated, which are then detected by the PD and finally Fourier transformed through the signal processor. When an external vibration is applied on the sensing fiber, a beat signal whose intermediated frequency *f*_0_ proportional to the distance of vibration position *x* is thereby produced, such that:

*x* = (*c*·*f*_0_)/(2*n*γ) (15)

where γ is the frequency sweep rate of the TLS. Thus, the vibration position can be achieved by analyzing the frequency spectrum. In addition, the SR in OFDR, ∆*z*, can be expressed as [132]:

∆*z* = *c*/(2*n*∆*F*) (16)

where Δ*F* is the frequency sweep range of TLS. Hence, in the OFDR system, the SR is solely determined by the frequency scan range of the laser. Due to its high SR and simple configuration, the OFDR technique can carry out strain and temperature measurements [133,134]. In recent years, dynamic measurement based on OFDR technique was also carried out. For instance, a time-resolved OFDR was demonstrated for vibration detection [135]. In the system, the local Rayleigh backscattering spectrum shift of the vibrated state with respect to that of the non-vibrated state was measured in time domain and the vibration frequency information was obtained by analyzing the Rayleigh backscattering spectra in optical frequency domain. The experimental results showed that a 10-cm SR over a 17-m sensing fiber length was achieved, with a measurable frequency range of 0–32 Hz. However, this measurement range was too short to be used in some applications. Afterwards, based on the correlation analysis of the OFDR signals, Ding et al. presented a novel method to achieve a space-resolved long-range vibration detection system. Firstly, the measured signals, non-vibrated state and vibrated state, in time domain were converted to spatial domain through FFT. Then, a sliding window with a width of Δ*x* was applied to both of the two signals and thereby the total spatial span was divided into several segments. Next, the cross-correlation algorithm was carried out at each segment to achieve the “non-similar level.” Finally, the vibration location could be obtained by detecting a striking change of the “non-similar level.” The vibration frequency information was extracted from the beat signal in spatial domain. The experimental results showed that the sensor was able to detect vibrations with a frequency up to 2 kHz over a range of 12 km, with an SR of 5 m [132]. However, this method was not without flaws; for instance, the requirement for measurement of vibrated state and non-vibrated states as well as the digitalization of cross-correlation was relatively sophisticated.

Afterwards, a dynamic OFDR system based on a fast scanning laser and coherent detection scheme was demonstrated for vibration and acoustic sensing [136]. Over an entire fiber length of 10 km, impulses and sinusoidal stretching were successfully detected. Then, a deskew filter method [137] was utilized to compensate the nonlinear phase of the TLS and the measurement range was extended to 40 km, with an SR of 6.7 m [138]. In these systems, the position and frequency of the vibration were obtained by the cross-correlation analysis of the beat signals between the vibrated state and non-vibrated state based on the amplitude extraction method. Then, a novel distributed quantitative vibration sensing technique based on phase extraction from time-gated digital OFDR, in which the phase noise from the laser source was greatly mitigated since the light frequency sweeping time was much shorter than the coherent time of the laser, was proposed [139]. The experimental results showed that the sensor had a measurement range of 40 km, SR of 3.5 m, measurable vibration frequency up to 600 Hz, and a minimal measurable vibration acceleration of 0.08 g.

### 2.3. Combination of Interferometric and Backscattering-Based Sensing Technology

**Figure 15:**
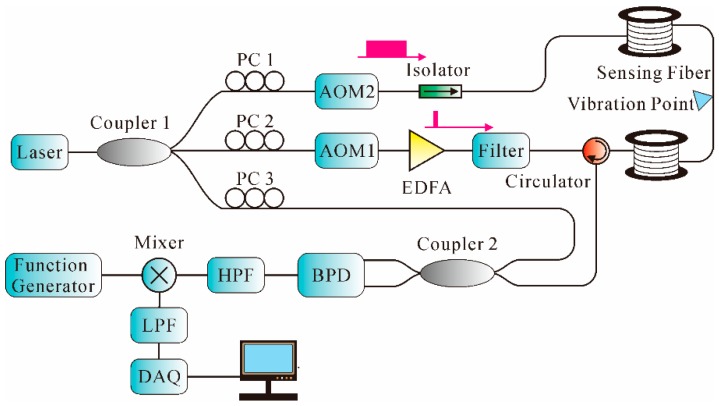
Experimental setup of distributed vibration sensing system merging the MZI and Φ-OTDR systems using modulated time-difference pulses, adapted from He et al. [141].

As we know, in conventional backscattering-based sensing systems, the detectable frequency response is determined by the pulse repetition rate. The increment of the pulse repetition rate improves the detected frequency range at the expense of a decreased fiber length. On the other hand, interferometric sensors offer wide frequency range measurement; however, the SR is relatively low. Thus, more and more studies have investigated the combination of interferometric and backscattering-based sensing technology in recent years. For instance, a modulated-pulse-based distributed vibration sensing method merging MZI and the Φ-OTDR system for high-frequency response and SR was demonstrated [140]. By carefully designing the modulated light pulses, a narrow pulse with high intensity and a wide pulse with low intensity within one period was generated. The modulated light pulses were launched into the FUT of an MZI and interfered with the reference light, while the Rayleigh backscattering light was collected through a circulator. Eventually, the vibration location and frequency information were obtained by Φ-OTDR and MZI, respectively. By using moving averaging and moving differential method, a 5-m SR was achieved over a 1064-m sensing fiber, with a lowest frequency response of 10 Hz and highest frequency response of 3 MHz. However, the Rayleigh backscattering light induced by the low-intensity pulse light could decrease the SNR of the system. Thus, another similar system that adopted two AOMs to generate narrow and wide pulses was demonstrated [141], using the arrangement shown in Figure 15.

As can be seen from Figure 15, two AOMs were adopted to generate narrow and wide pulses, respectively, on both ends of a sensing fiber with a time difference. Narrow pulses were used to generate Rayleigh backscattering light, which located the vibration point, while the wide pulses interfered with the reference light as an MZI to obtain the frequency response. By using a TDM strategy, the signal intensity of the interferometer was improved and the highest detectable frequency increased to 6.3 MHz along a 1150-m sensing fiber. However, it was difficult to control the timing sequence and process the data.

**Figure 16:**
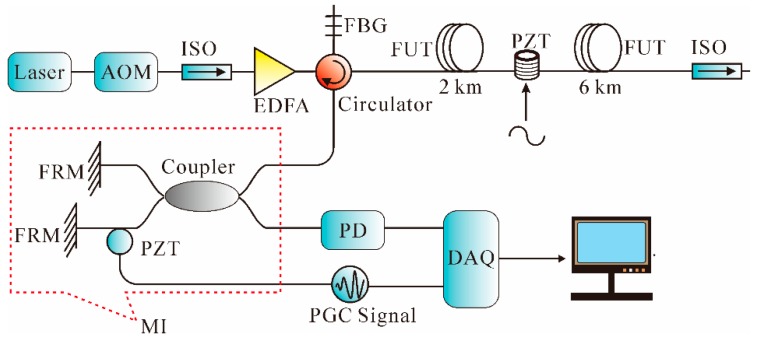
Experimental setup of a distributed vibration sensing system merging the MI and Φ-OTDR systems based on the PGC algorithm, adapted from Fang et al. [142].

Later, in [142], a novel scheme combining Φ-OTDR and MI was proposed based on the PGC algorithm using the arrangement shown in Figure 16. The Rayleigh scattering light interfered through an unbalanced MI (6 m fiber delay between two arms) to generate the interference light, which was modulated by a sinusoidal PGC signal. Then, the output of PD, which contained the location information, and a PGC signal, which contained the phase information, were both sampled by the DAQ card. In the experiment, a 2-V sinusoidal signal with 120 Hz frequency was applied on a PZT cylinder at the position of 2 km. The experimental results showed that the vibration signal was well recovered.

Afterwards, a novel Φ-OTDR system merged with MZI for wide frequency response and high location accuracy was reported [143]. In the system, the WDM strategy was adopted. One laser was deployed to build the Φ-OTDR architecture, which detected location information of the vibration, while the other laser was deployed to build the MZI architecture, which detected the frequency information of the vibration. The experimental results showed that the maximum detected response was almost 50 MHz and the SR was 20 m along a 2.5-km sensing fiber.

In addition, the concept of quantifying strain by adding an interferometer at the end of a coherent Rayleigh backscattering-based sensing system was proposed and demonstrated by Posey et al. [144]. The backscattering Rayleigh light from the FUT was collected by a MZI. Then, through two unbalanced arms of MZI, the backscattering signals interfered from two separate scattering regions of FUT. When an external strain was applied between the scattering regions, the changing phase and hence the applied strain was measured. Based on this concept, a distributed fiber-optic dynamic strain sensor, which was capable of quantifying multiple dynamic strain perturbations simultaneously, was proposed [145]. The experimental results showed that over a 1-km sensing fiber, the minimum detectable strain perturbation of the sensor was 80 nε, with an SR of 2 m and a frequency range of 500–5000 Hz.

### 2.4. Summary of Distributed Fiber-Optic Vibration Sensors

A brief list of performance summary of distributed fiber-optic vibration sensors is shown in Table 1, in which the detection method, research group, sensing distance, SR/position accuracy, vibration frequency, and multi-point capability are all presented.

## 3. Applications

In the past decades, distributed fiber-optic vibration sensors have found extensive applications. They have been utilized in health monitoring of civil infrastructures, perimeter security protection, borehole seismic applications, etc. Many efforts have been made to accomplish these demanding needs with distributed fiber-optic vibration sensing technologies.

### 3.1. Structural Health Monitoring (SHM)

In the past decades, the demand for SHM has increased dramatically in our society. Safety monitoring of civil infrastructures, such as bridges, highways, tunnels, oil and gas pipelines, high-voltage power cables, etc., is important to ensure social stability and rapid economic development. However, due to the wide geographic distribution of these infrastructures, the application of conventional methods that can only provide point measurement is severely limited. On the other hand, distributed fiber-optic sensor technology is one of the most promising candidates among the numerous sensing technologies that are suitable for the SHM field [148]. This is due to the inherent fiber optic properties such as light weight, small size, non-corrosive nature, immunity to EM interference, distributed sensing capability, etc. [149]. Thus, distributed fiber-optic sensing technology shows considerable potential for these types of applications, such as detection of pipeline leakage [150] and cracks of civil infrastructures [90], etc.

The distributed fiber-optic vibration sensors are widely investigated in pipeline leakage detection. As we know, pipelines are an important part of the infrastructure in our society and are indispensable for the transportation of water, gas, oil, and various other products [151]. Long-distance oil/gas pipelines are often buried several meters underground and are vulnerable to natural or man-made damage, such as earthquakes, construction work executed by engineering vehicles on the line, and pipeline drilling and oil stealing by lawbreakers, which may cause pipeline vibration or even leakage. Conventional methods for judging pipeline leakage rely on manual inspection or detecting devices such as pressure sensors, chemical sensors, thermal sensors, acoustic sensors, etc. These methods are liable to be influenced by the quality of the material transported and other factors. Besides, warning of leakage comes only after the leakage occurs, which may cause loss of life and property along with environmental pollution. Strong interest exists in novel sensors, which can realize large-scale measurement and even perform pre-warning monitoring and locating when the events are taking place. Fortunately, distributed fiber-optic vibration sensors are ideal for these applications due to their fully distributed manner that can provide vibration information along the whole fiber length, which can go up to several hundred kilometers, as well as the abilities of pre-warning, high sensitivity, accurate localization, etc. For instance, in [152] a distributed fiber-optic vibration sensor for detection and location of the pipeline leakage is proposed. A pipe with high-pressure nitrogen inside is utilized to simulate the actual high-pressure pipelines in practical application. The sensing fiber is fixed on the outside of pipelines by epoxy resin. In the experiment, leakage position under different distance and pressure is measured and the test results show the capability of the system to detect pipeline leakage. Commercially available systems such as Silixa’s intelligent pipeline surveillance system (iPSS™) can provide pipeline integrity management including real-time leakage detection and threat identification, etc.

Crack detection of civil infrastructures is also an important area in the field of SHM. Due to the advantages of large-scale monitoring, high sensitivity, and dynamic monitoring capacity, distributed fiber-optic vibration sensors attract more and more research attention in the field of SHM. For instance, in [90], a laboratory test demonstrating the feasibility of a distributed fiber-optic vibration sensor in bridge crack detection is reported. In the experiment, the acoustic emission of cracks in concrete or steel bridges is emulated in the laboratory. By using the developed distributed fiber-optic vibration sensor, broadband acoustic frequency components generated by the simulated crack actions can be successfully detected and located. The experimental results show the potential of distributed fiber-optic vibration sensors in practical health monitoring of various civil infrastructures.

However, more improvements are needed before this technology will be ready for practical application, such as processing of weak detection signals, enhancing system reliability and positioning accuracy in a complex environment, implementing high-speed and real-time signal processing, etc. If these issues are solved, distributed fiber-optic vibration sensing technique can become a powerful tool in the field of SHM and may have huge social benefits and broad application prospects in the future.

### 3.2. Perimeter Security

In important sites such as airports, seaports, military bases, nuclear facilities, and electrical power plants, perimeter security [153] systems that serve as a concealed “electronic fence” are widely used to avoid equipment destruction and protect property from being vandalized. The traditional patrol-based method suffers from limitations in terms of scope, continuity, and reliability. Distributed fiber-optic vibration sensors are very sensitive and can detect small disturbances and perform pre-warning when the perimeter line is disturbed. Nowadays, distributed fiber-optic vibration sensing techniques are more and more widely used in perimeter security applications. For example, a national border monitoring system has been in operation along the 220 km long national borderline in the north of China since July 2012 [154]. Industrial application systems produced by OptaSense can provide real-time monitoring of up to 40-km lengths so that military personnel are constantly updated on potential movements, behaviors, and any activity along the fiber length.

In addition, the distributed fiber-optic vibration sensors can also be utilized as a fiber fence [155], physical security system [156], disturbance sensor [157,158], and intrusion sensor [159], etc. Among them, distributed fiber-optic intrusion sensors have been extensively investigated in past decades due to their superiority over conventional intrusion sensors, which can only be individually used in limited areas and often have poor concealment. For instance, in [74], a distributed fiber-optic vibration sensor for perimeter intrusion detection was demonstrated. Field tests were performed and various tests on a sensing fiber hung on a fence and a sensing mat were carried out, such as the system stability and intrusion detection under environmental disturbance. Field tests show that the system has operated stably for six months, with a false alarm rate of less than 4%. In [156], a physical security system utilizing distributed fiber-optic vibration sensing technology was reported. In field tests, the sensing fiber was installed in the perimeter fence of an important facility. The act of an individual climbing up and down the fence is repeated several times a day. Field tests show that though false alarms occur about once per month, the physical security system functions well with a detection rate of 100%.

### 3.3. Other Related Applications

In addition to applications in the field of SHM and perimeter security, distributed fiber-optic vibration sensors also show considerable potential in the field of borehole seismic applications for its capability of profiling the whole fiber link in a single measurement [160]. In the oil and gas industry, seismic techniques, especially borehole seismology, are widely utilized for the characterization of underground structure and monitoring of production processes [161]. Recently, many efforts have been made to apply distributed fiber-optic vibration sensing technology for borehole seismic acquisition. For instance, in [162], a distributed fiber-optic vibration sensor conveyed on wireline cable is proposed for vertical seismic profiling. The test results show that the sensor is able to record the entire seismic profile with a single shot of the seismic source. Industrial application systems produced by OptaSense can offer oilfield services including vertical seismic profiling, hydraulic fracture profiling, production flow monitoring, etc.

**Figure 17:**
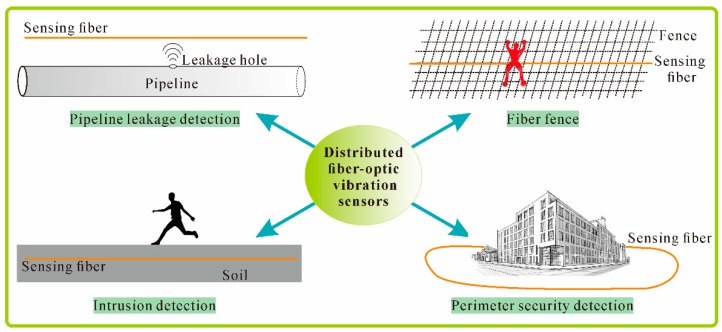
Several typical applications of distributed fiber-optic vibration sensors.

In summary, distributed fiber-optic vibration sensing technology can in a relatively cost-effective manner provide perimeter security at various places, SHM for many civil infrastructures, and borehole seismic acquisition in the oil and gas industry, etc. With the superior ability to simultaneously provide measurement information along tens or even hundreds of kilometers, distributed fiber-optic vibration sensors play a more and more important role in vibration monitoring and analysis area. Figure 17 illustrates several typical applications of distributed fiber-optic vibration sensors, including pipeline leakage detection, intrusion detection, perimeter security detection, fiber fences, etc.

## 4. Conclusions

Distributed fiber-optic vibration sensing technology is able to provide fully distributed vibration information along the entire fiber link, and thus external vibration signals from arbitrary point can be detected and located. Optical parameters such as light intensity, phase, polarization state, and light frequency will change when external vibration is applied on the sensing fiber. Various technologies of distributed fiber-optic vibration sensing are reviewed, from interferometric sensing technology, such as Sagnac, MZI, and MI, to backscattering-based sensing technology, such as Φ-OTDR, POTDR, BOTDA, BOCDA, and OFDR, as well as some combination of interferometric and backscattering-based sensing technology. Each technique has its own edge in terms of cost, complexity, and performance, though special concern is given to the Φ-OTDR sensing technology. Nowadays, it is the most suitable technique and can be used for multiple vibrations monitoring in a comparatively cost-effective manner over a large monitoring distance.

At the end, the applications of distributed fiber-optic vibration sensors are summarized. Firstly, this technology attracts a significant amount of research attention in the SHM field such as detection of oil and gas pipeline leakage and cracks of various civil infrastructures, etc. Secondly, distributed fiber-optic vibration sensors exhibit impressive advantages in the perimeter security fields. They have been successively used as national border monitoring system, fiber fence, physical security system, perimeter intrusion detection system, etc. Finally, distributed fiber-optic vibration sensing also shows considerable potential in the field of borehole seismic applications.

Overall, the distributed fiber-optic vibration sensing technique provides great advantages of large-scale monitoring, good concealment, excellent flexibility, and immunity to EM interference, and thus shows considerable potential for a variety of practical applications and may have huge social benefits in the future.

## Figures and Tables

**Table 1:** Performance summary of distributed fiber-optic vibration sensors.

Methods	Group	Distance (km)	SR/ Position Accuracy (m)	Frequency? Yes/No (Hz)	Multi-point? Yes/No	Year	Reference
Sagnac	University of Kansas, USA.	0.18	1	Y	N	1996	[42]
	Rand Afrikaans University, South Africa	0.2	-	Y	N	1998	[43]
	Virginia Polytechnic Institute and State University, USA	0.8	-	Y	N	1996	[44]
	University of Southampton, England	40	100	Y	Y	2001	[49]
	Huazhong University of Science and Technology, China;	41	100	Y	Y	2014	[51]
	Fudan University, China	25	59	Y	Y	2008	[50]
		150	-	Y	Y	2015	[52]
MZI	Huazhong University of Science and Technology, China	1.01	38	Y	Y	2008	[59]
	Beihang University, China	20	206	Y	N	2009	[61]
	Tianjin University, China	50	±50	Y	N	2008	[55]
		2.25	±20	Y	N	2015	[64]
	Southeast University, China	10	-	Y	N	2013	[146]
	Beijing Jiaotong University, China	2	75	Y	N	2015	[65]
	Jinan University, China	320	31	9 M	Y	2014	[34]
MI	Beijing University of Posts and Telecommunications, China	4.012	±51	Y	N	2011	[70]
Sagnac + MZI	Rand Afrikaans University, South Africa	0.2	5	Y	N	1998	[73]
Sagnac + MI	University of Pretoria, South Africa	0.2	±4	Y	N	1997	[77]
	Military University of Technology, Poland	6	40	Y	N	2007	[78]
Φ-OTDR	Texas A&M University, USA	12	1000	N	Y	2005	[87]
		12	200	N	Y	2005	[88]
		19	200	N	Y	2007	[89]
	University of Ottawa, Canada	1.2	5	1 k	Y	2010	[90]
		0.2	1	~2.25 k	Y	2011	[91]
		1	0.5	8 k	Y	2012	[102]
	Chongqing University, China	1	~3	N	Y	2013	[104]
	Faculdade de Ciencias da Universidade do Porto, Portugal	1.25	5	39.5 k	Y	2013	[92]
		125	10	250	Y	2014	[98]
		125	10	380	Y	2015	[99]
	University of Electronic Science and Technology of China, China	131.5	8	375	Y	2014	[100]
		175	25	Y	Y	2014	[101]
		2.7	3.7	∼18 k	Y	2015	[107]
	University of Ottawa, Canada; Shandong University, China	0.68	1	0.6 M	Y	2014	[93]
	University of Shanghai for Science and Technology, China	44	5	N	Y	2014	[147]
	Nanjing University, China	24.61	10	Y	Y	2015	[108]
		9	2	1 K	Y	2015	[109]
POTDR	University of Ottawa, Canada	1	10	5 k	Y	2008	[112]
	University of Mons, Belgium	~0.47	5	N	N	2012	[113]
	Nanjing University, China	4	10	610	N	2013	[114]
		7	10	Y	Y	2013	[118]
		10	10	Y	N	2015	[116]
BOTDA	Lehigh University, USA; Beihang University, China	0.168	0.625	Y	Y	2011	[121]
	Tel-Aviv University, Israel	0.085	1.5	Y	Y	2011	[122]
		0.1	1.3	Y	Y	2012	[124]
	Harbin Institute of Technology, China	0.05	0.2	50	Y	2013	[125]
BOCDA	The University of Tokyo, Japan	0.02	0.1	200	Y	2007	[128]
	Chung-Ang University, Korea; The University of Tokyo, Japan	0.1	0.8	1.3	Y	2011	[129]
OFDR	Chongqing University, China	0.017	0.1	32	Y	2012	[135]
	Tianjin University, China	12	5	2 k	Y	2012	[132]
		40	6.7	Y	Y	2016	[138]
	Tel-Aviv University, Israel	10	40	Y	Y	2014	[136]
	Shanghai Jiao Tong University, China	40	3.5	600	Y	2015	[139]
Φ-OTDR + MZI	Chongqing University, China; University of Ottawa, Canada	1.064	5	3 M	N	2013	[140]
		1.150	5	6.3 M	N	2013	[141]
	University of Southampton, Southampton, UK	1	2	500–5 k	Y	2013	[145]
	Tian Jin University, China	2.5	20	50 M	N	2015	[143]
Φ-OTDR + MI	Chinese Academy of Sciences, Beijing, China	10	6	Y	N	2015	[142]
